# Fish-Specific Duplicated *dmrt2b* Contributes to a Divergent Function through Hedgehog Pathway and Maintains Left-Right Asymmetry Establishment Function

**DOI:** 10.1371/journal.pone.0007261

**Published:** 2009-09-30

**Authors:** Sha Liu, Zhi Li, Jian-Fang Gui

**Affiliations:** State Key Laboratory of Freshwater Ecology and Biotechnology, Institute of Hydrobiology, Chinese Academy of Sciences, Graduate School of Chinese Academy of Sciences, Wuhan, China; Harvard University, United States of America

## Abstract

Gene duplication is thought to provide raw material for functional divergence and innovation. Fish-specific *dmrt2b* has been identified as a duplicated gene of the *dmrt2a/terra* in fish genomes, but its function has remained unclear. Here we reveal that Dmrt2b knockdown zebrafish embryos display a downward tail curvature and have U-shaped somites. Then, we demonstrate that Dmrt2b contributes to a divergent function in somitogenesis through Hedgehog pathway, because Dmrt2b knockdown reduces target gene expression of Hedgehog signaling, and also impairs slow muscle development and neural tube patterning through Hedgehog signaling. Moreover, the Dmrt2b morphants display defects in heart and visceral organ asymmetry, and, some lateral-plate mesoderm (LPM) markers expressed in left side are randomized. Together, these data indicate that fish-specific duplicated *dmrt2b* contributes to a divergent function in somitogenesis through Hedgehog pathway and maintains the common function for left-right asymmetry establishment.

## Introduction

Gene duplication is thought to be the primary source of new genes. Many teleost fish, including zebrafish, experience an additional genome wide duplication event [1]. Since then, many of the duplicated genes have been lost, but a substantial percentage of the duplicates have been retained, and functional divergence has occurred in some duplicates [1], [2]. Recently, to identify some differentially expressed genes in early embryogenesis, two kinds of SMART cDNAs were respectively synthesized from the mature eggs and gastrula embryos, and the gastrula embryo SMART cDNA library was constructed in *Carassius auratus gibelio* [3], [4], [5], [6], [7]. Following this program, many differentially expressed genes at gastrula stage were screened [5], and some of them were characterized and functionally analyzed [8], [9]. Significantly, a fish-specific duplicated gene *dmrt2b* was identified in *dmrt* gene family from *Carassius auratus gibelio* [4].

*dmrt2*, *dsx* and *mab-3* related transcription factor 2, is a member of a gene family of putative transcription factors. These transcription factors share a highly conserved zinc-finger-like DNA-binding domain (DM domain) which is implicated in sex determination [10]. However, recent studies show that the family genes function not only in sex determination, but also in embryonic development [11]. In zebrafish, *dmrt2* was originally called *terra* [12]. *dmrt2b* is another duplicated copy of the *dmrt2* in the genome, and *dmrt2a*/*terra* and *dmrt2b* have been designated for distinguishing them. Interestingly, they share similar expression pattern in the muscle-related tissues, but *dmrt2b* is also expressed in branchial arches, which is different from *dmrt2a* [13]. The preliminary data suggested that *dmrt2b* and *dmrt2a* might have both common functions and divergent functions. In zebrafish, *dmrt2a* was found to be a left-right (LR) asymmetry gene required for left-right synchronization of the segmentation clock [14], but the LR asymmetry defect has not been observed in mouse embryos lacking *Dmrt2* function. In contrast with the LR asymmetry requirement of *dmrt2a* in zebrafish, the loss of *Dmrt2* in mouse leads to embryonic somite patterning defects [15]. Intriguingly, a previous study in zebrafish observed that overexpression of *dmrt2a* induced rapid apoptosis in the mesoderm, and suggested that Dmrt2a might be involved in somitogenesis of vertebrates [12]. The seemingly contradictive data propose that functional divergence might have occurred between the two duplicated genes of *dmrt2a* and *dmrt2b*. However, exact function of the fish-specific duplicated gene *dmrt2b* has remained unknown. This study aims to reveal the biological functions of *dmrt2b* and to examine the possible underlying mechanism and signal pathway by using morpholino-mediated knockdown strategy in the model animal zebrafish.

## Results

### Molecular characterization and expression pattern of Dmrt2b during embryogenesis

**Figure 1:**
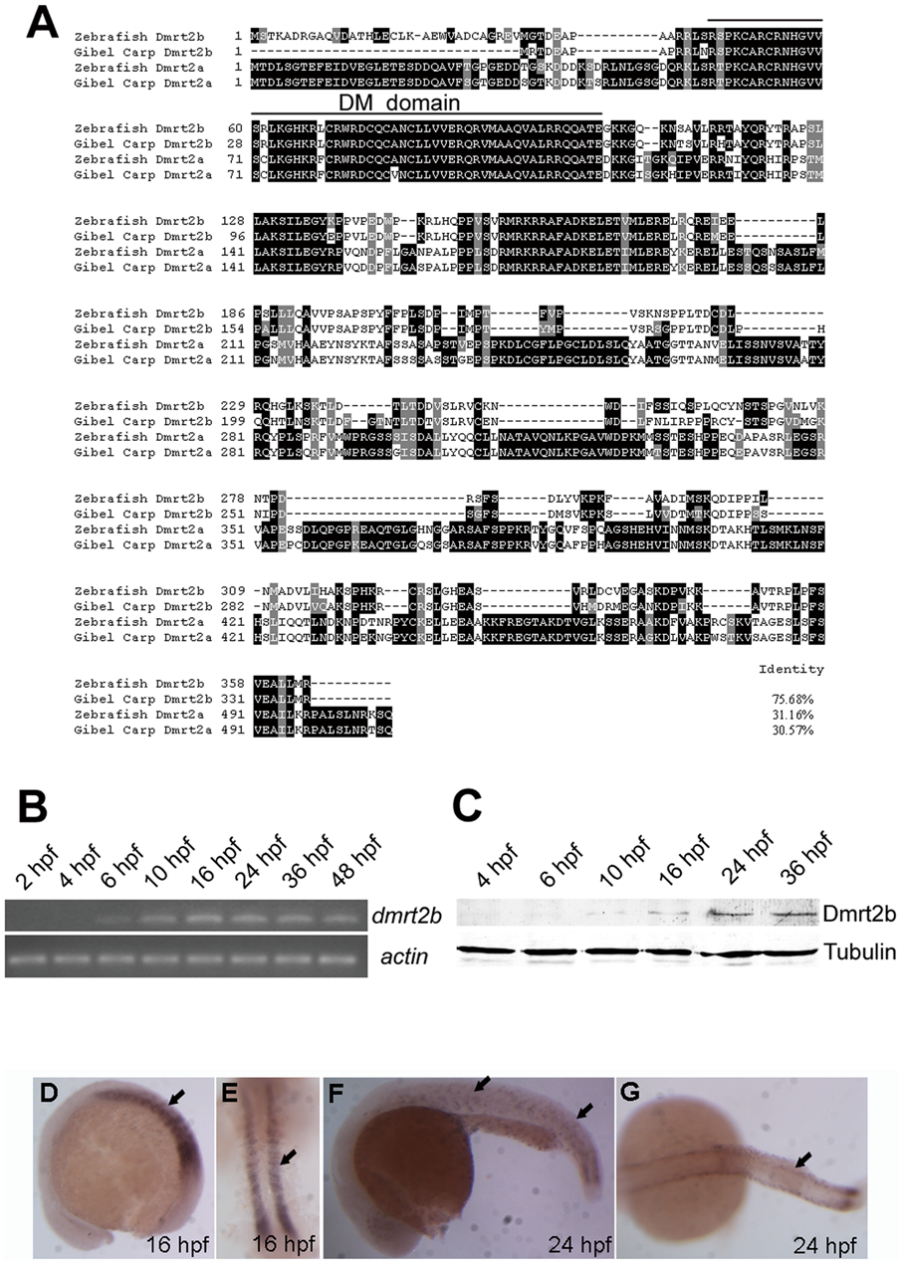
Molecular characterization and expression pattern of zebrafish Dmrt2b during embryogenesis. (A) Amino acid alignment of zebrafish Dmrt2b with gibel carp Dmrt2b, gibel carp Dmrt2a and zebrafish Dmrt2a. Similar and identical amino acids are highlighted in grey and black boxes. The line indicates the DM domain. (B) RT-PCR detection of *dmrt2b* in zebrafish embryonic development stages, and beta-actin mRNA as the control. (C) Western blot detection of Dmrt2b during the zebrafish embryo development, and Tubulin was used for the control. (D-H) Whole-mount *in situ* hybridization detection of *dmrt2b* on somitogenesis embryos as indicated stage. The arrows indicate positive signals in the somites.

As a new member of *dmrt* gene family, *dmrt2b* was firstly cloned in gibel carp (*Carassius auratus gibelio*) (GenBank accession number: EF029082). Database searches revealed the closest homologue in zebrafish (*Danio rerio*) (GenBank accession number: NM_001079976). The DM domain of zebrafish Dmrt2b is 100% identical to that of gibel carp Dmrt2b, and has 94.7% and 93.0% identities to that of zebrafish and gibel carp Dmrt2a respectively. Significantly, the full-length amino acid sequence of zebrafish Dmrt2b has only 31.16% and 30.57% identities to zebrafish and gibel carp Dmrt2a, although it has 75.68% identity to gibel carp Dmrt2b (Figure 1A). The significant sequence divergence between Dmrt2b and Dmrt2a implicates the potential occurrence of functional divergence.

Subsequently, the expression pattern of zebrafish *dmrt2b* during embryogenesis was analyzed by RT-PCR, Western blotting and whole-mount *in situ* hybridization. As shown in Figure 1B and Figure 1C, the *Dmrt2b* transcription is initiated from around shield stage at 6 hpf, and kept at a basic stable level from bud stage at 16 hpf to the hatched larvae stage at 48 hpf. And, the Dmrt2b protein is expressed after bud stage at 10 hpf, and the abundant expression level is detected after 24 hpf of embryogenesis. Figure 1D–G shows *in situ* mRNA distribution of *dmrt2b* during embryogenesis. Significantly, *dmrt2b* is specifically expressed in somites during somitogenesis.

### Dmrt2b knockdown leads to defects in somitogenesis and reduces target gene expression of Hedgehog signaling

**Figure 2:**
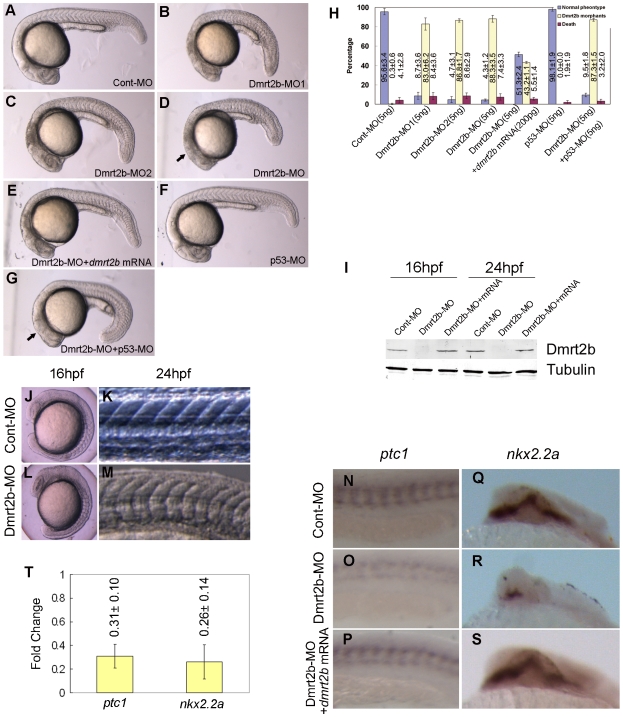
Dmrt2b morphants display defects in somitogenesis and Hedgehog signaling. (A–G) Morphology of 24 hpf embryos injected with Cont-MO (A), Dmrt2b-MO1 (B), Dmrt2b-MO2 (C), Dmrt2b-MO (D), Dmrt2b-MO+*dmrt2b* mRNA (E), p53-MO (F), Dmrt2b-MO+p53-MO (G). The arrows indicate the off-target cell death in Dmrt2b-MO morphant and the reduced cell death in the Dmrt2b-MO+p53-MO morphant. (H) The statistical data of three independent experiments on Dmrt2b knockdown, *dmrt2b* mRNA rescue and p53 MO co-injection. Results are represented as mean±SD of three separate experiments. (I) Western blot detection of Dmrt2b knockdown during embryogenesis. The protein extracts from embryos (16 hpf and 24 hpf) were analyzed by Western blot using the polyclonal anti-Dmrt2b antibody. A band of about 41 KD was not detected in Dmrt2b morphants. The picture represents typical result from three separate experiments. (J–M) Dmrt2b morphant exhibits U-shape somites. Morphology of embryos injected with Cont-MO display the typical ‘chevron’ shape (J, K). Morphology of embryos injected with Dmrt2b-MO display the U-shape (L, M). Whole-mount *in situ* hybridization of *ptc1*(N, O, P) (Anterior is left) and *nkx2.2a*(Q, R, S) (Anterior is top) in embryos injected with Cont-MO (N, Q) or Dmrt2b-MO (O, R) and embryos co-injected with Dmrt2b-MO with *dmrt2b* mRNA (P, S) at 24 hpf. (T) qPCR analysis of the expression changes of *ptc1* and *nkx2.2a* in 24 hpf embryos injected with Cont-MO or Dmrt2b-MO. Results represent mean±SD of three separate experiments.

Abundant expression of *dmrt2b* in somites implies its significant functions in zebrafish embryo development. To assess the functions, we undertook loss-of-function experiments in zebrafish by morpholinos. We utilized two non-overlapping antisense morpholino oligonucleotides (MOs), Dmrt2b-MO1 and Dmrt2b-MO2, which designed to block translation by binding to 25 bases of the 5′ UTR upstream of the start codon (Supplementary Information, Figure S1A), because the translation-blocking morpholino was demonstrated to be a powerful tool for studying the effects of near-total loss of function during early stages of development [16]. Western blot detection showed a predicted size protein band in the Cont-MO embryos at 24 hpf, whereas the corresponding protein band was notably reduced in the embryos injected with Dmrt2b-MO1(5 ng per embryo) and Dmrt2b-MO2 (5 ng per embryo) respectively, and no any signal was observed in the embryos co-injected with Dmrt2b-MO1 (2.5 ng) and Dmrt2b-MO2 (2.5 ng) (Supplementary Information, Figure S1B). Morphological observation displayed obvious embryonic development defects in the Dmrt2b morphants. As shown in Figure 2, in comparison with normal embryos injected with 5 ng Cont-MO (Figure 2A), the morphant embryos injected with 5 ng Dmrt2b-MO1 or 5 ng Dmrt2b-MO2 exhibit an abnormal ventrally curved body shape, shot trunk and U-shaped somites at 24 hpf (Figure 2B,C), and when the two non-overlapping antisense MOs (2.5 ng each, totally 5 ng) were jointly injected, the morphant embryos exhibit similar phenotypes and even more severe defects (Figure 2D). Because co-injection of multiple targeting MOs was known to exert maximal effects [17], [18], [19], we combined Dmrt2b-MO1 and Dmrt2b-MO2 as Dmrt2b-MO for subsequent analysis. To confirm specificity of the embryonic defects, we performed rescue experiments in which we co-injected the Dmrt2b-MO with the *in vitro* transcribed *dmrt2b* mRNA. Significantly, the co-injected *dmrt2b* mRNA successfully rescued the embryonic defects in the morphants (Figure 2E). Since some off-targeting effects of morpholinos were found to be mediated through p53 activation, and p53-MO can attenuate the off-targeting effects [20], [21], we also checked the embryonic effects of Dmrt2b-MO and p53-MO co-injection. In comparison with normal embryos injected with p53-MO (Figure 2F), the co-injection embryos still exhibited the ventrally curved body shape, shot trunk and U-shaped somites, identically to the Dmrt2b morphants, whereas only cell death was reduced in the anterior and trunk regions (Figure 2G) compared with the Dmrt2b morphant (Figure 2D). The data indicated that the cell death observed in anterior and trunk regions of the Dmrt2b morphant appeared to be due to the off-target effects, because p53-MO co-injection could rescue the defect, and the defects in somitogenesis is specific to Dmrt2b knockdown, because they could not be rescued by p53-MO co-injection, which confirmed the specificity of the embryonic defects in the Dmrt2b morphant embryos. Figure 2H shows the statistical data of three independent experiments on the Dmrt2b knockdown, *dmrt2b* mRNA rescue and p53-MO co-injection.

Furthermore, the ability of these MOs to block the endogenous *dmrt2b* translation was examined by Western blot. As shown in Figure 2I, the Dmrt2b protein was not detected in the morphants at 16 hpf and 24 hpf, whereas a about 41kD Dmrt2b protein band was observed in the Cont-MO and mRNA rescue embryos. The data indicate that the Dmrt2b-MO specifically blocks the *dmrt2b* mRNA translation, and that the morphant defects are resulted from the specific translation blocking of *dmrt2b* mRNA.

A detailed morphological observation for Dmrt2b morphants revealed similar common traits in Hedgehog pathway mutants, such as a curled tail, U-shaped somite boundaries and slow muscle defect [22], [23], [24], [25], [26], [27]. For example, in comparison to the typical ‘chevron’ shape (Figure 2J, K) in control embryos, the Dmrt2b morphants display severe U-shaped somites at 16 hpf (Figure 2L) and at 24 hpf (Figure 2M). To find further evidence that Dmrt2b is involved in Hedgehog signal pathway, we first checked the expression affection of *patched1* (*ptc1*), a direct transcriptional target of the Hh pathway [28]. In comparison with high expression in somites of Cont-MO embryos (Figure 2N), an obvious down-regulation of *ptc1* transcript was observed in the Dmrt2b morphants (23/27 embryos) at 24 hpf (Figure 2O). In embryos co-injected with Dmrt2b-MO and *dmrt2b* mRNA, low expression of *ptc1* was rescued (21/25 embryos) at 24 hpf (Figure 2P). To reveal the signal point at which Dmrt2b exerts its activity on the Hh signaling cascade, we also examined the expression of the Hh target gene *nkx2.2a* in the developing brain [29]. As shown in Figure 2Q–S, Dmrt2b-MO causes a notable reduction of *nkx2.2a* in the brain (22/25 embryos), and co-injection with *dmrt2b* mRNA could rescue the phenotype (19/24 embryos). Quantification analysis through qPCR further confirmed the down-regulated changes of *nkx2.2a* and *ptc1* expression (Figure 2T).

**Figure 3:**
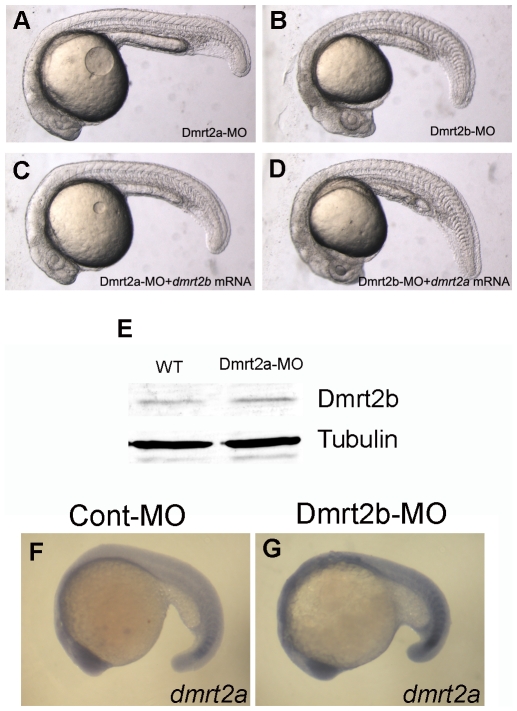
Dmrt2a and Dmrt2b can not compensate for each other. (A–D)Morphology of 24 hpf embryos injected with Dmrt2a-MO (A), Dmrt2b-MO (B), Dmrt2a-MO+*dmrt2b* mRNA (C), Dmrt2b-MO+*dmrt2a* mRNA (D). (E) Western blot assay showing Dmrt2b expression level in the embryos injected with wild type (WT) and Dmrt2a-MO. (F, G) The expression of *dmrt2a*(*terra*) was not affected in Dmrt2b morphants. Lateral views of embryos at 18 somites stage.

Considering sequence similarity and functional redundancy of Dmrt2a and Dmrt2b, we additionally tested the ability whether Dmrt2a could rescue Dmrt2b knockdown and *vice versa*. As shown in Figure 3, both Dmrt2a and Dmrt2b morphant defects (Figure 1A, B) could not be rescued by *dmrt2b* mRNA (Figure 3C) and *dmrt2a* mRNA respectively (Figure 3D). Then we analyzed the protein level of Dmrt2b in the Dmrt2a-MO injected embryos, and no any notable change of Dmrt2b expression was observed in the Dmrt2a morphants (Figure 3E). We also examined the expression of *dmrt2a* in the Dmrt2b morphants by whole-mount *in situ* hybridization, and no any change was not detected (Figure 3F and G). The data indicate that the loss-of-function of Dmrt2b leads to notable defects in somitogenesis and reduces target gene expression of Hedgehog signaling. And, the defects do not affect the expression of *dmrt2a* and Dmrt2a and Dmrt2b can not compensate the function for each other when they are knocked down.

### Dmrt2b is involved in slow muscle development through Hedgehog signaling

**Figure 4:**
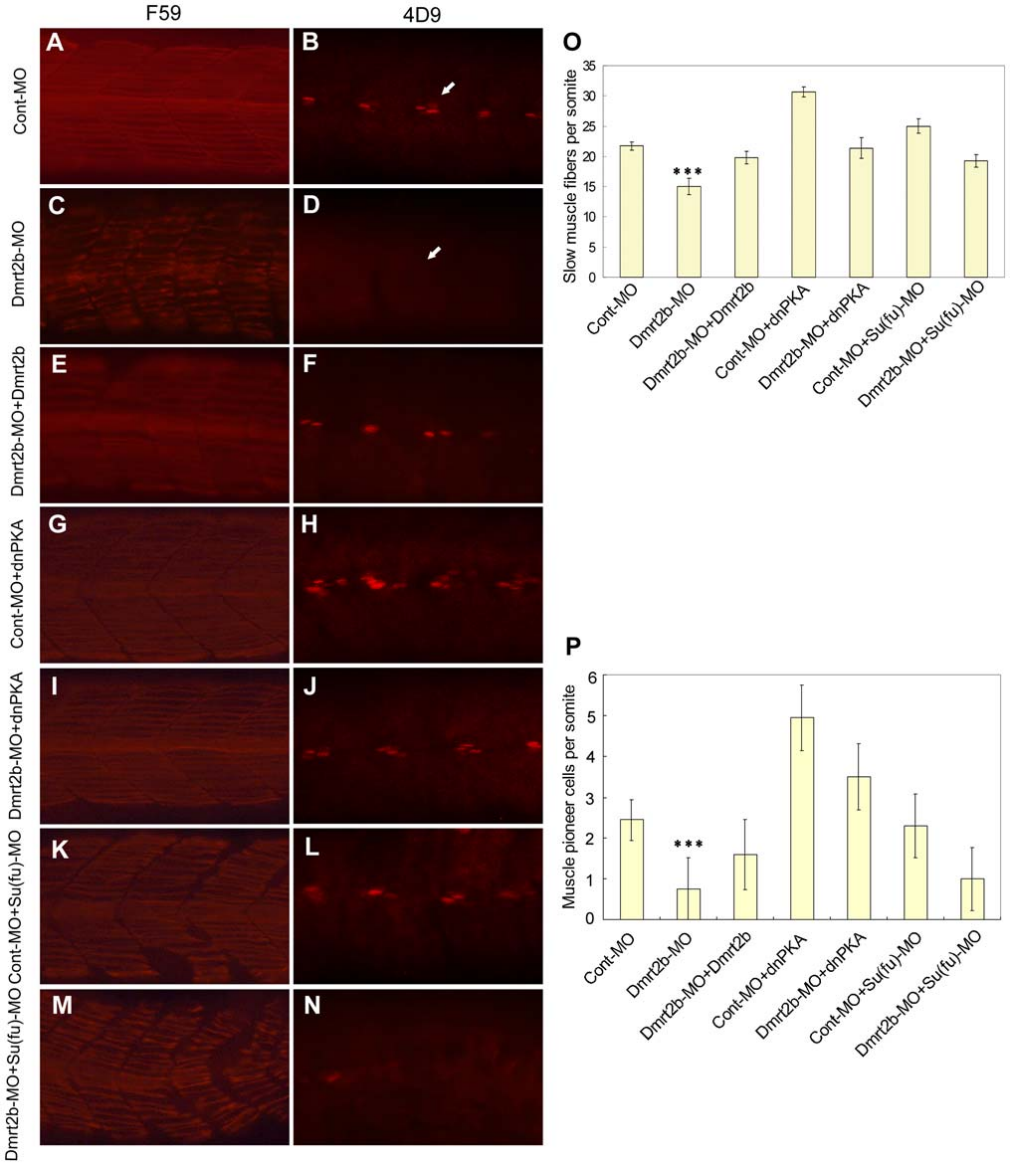
Dmrt2b is involved in slow muscle development through Hedgehog signaling. Analysis of slow muscle development by whole-mount immunostaining of 26 hpf embryos with antibodies labeling slow MyHC (F59) (A, C, E, G, I, K, M) and engrailed muscle pioneer cells (4d9) (B, D, F, H, J, L, N). Lateral view of embryos injected with Cont-MO (A, B), Dmrt2b-MO (C, D), embryos co-injected with Dmrt2b-MO+*dmrt2b* (E, F), Cont-MO+*dnPKA* (G, H), Dmrt2b-MO+*dnPKA* (I, J), Cont-MO+Su(fu)-MO (K, L), Dmrt2b-MO+Su(fu)-MO (M, N). White arrows in 4d9 panel indicate reduction of engrailed staining in the knockdown embryos compared to controls. All images show the somites over the yolk extension. Anterior is left in all images. (O) Quantitative analysis of slow muscle fiber number per somite in embryos at 26 hpf, injected as indicated. Data represent average±SD. *** indicates significance of *p*<0.0001 for Dmrt2b-MO vs. Cont-MO, Dmrt2b-MO+*dmrt2b*, Dmrt2b-MO+*dnPKA* and Dmrt2b-MO+Su(fu)-MO. n = 20 to 38 embryos per condition. (P) Quantitative analysis of muscle pioneer cells number per somite in embryos at 26 hpf, injected as indicated. Data represent average±SD. *** indicates significance of *p*<0.0001 for Dmrt2b-MO vs. Cont-MO, Dmrt2b-MO+*dmrt2b* and Dmrt2b-MO+*dnPKA*. n = 20 to 36 embryos per condition.

To assess the role of Dmrt2b in slow muscle development, we further analyzed the expression affect of Dmrt2b knockdown on slow muscle markers by using F59 antibody which labels slow myosin heavy clain (MyHC) in zebrafish [30] and 4D9 antibody which recognizes the *engrailed* protein in the nuclei of muscle pioneer cells. As shown in Figure 4, in comparison with normal superficial slow muscle fibers visualized by F59 antibody (Figure 4A) and Engrailed-expressing muscle pioneers stained by 4D9 antibody (Figure 4B) in Cont-MO embryos, the differentiation of slow muscle cells is severely perturbed in the morphant embryos. Especially, the numbers of superficial slow muscle fibers are obviously reduced (Figure 4C), and the Engrailed-expressing muscle pioneers are inhibited (Figure 4D) in the morphant embryos at 26 hpf. Significantly, the perturbed phenotypes can be rescued by co-injection of *dmrt2b* mRNA (Figure 4E, F). These data demonstrate that embryos with reduced Dmrt2b expression are unable to respond effectively to Hh signals during zebrafish segmentation, resulting in significant impairment in slow muscle development.

PKA is a negative regulator of the Hh pathway. As described previously [31], [32], when the dominant negative *PKA* (*dnPKA*) mRNA is injected into Cont-MO embryos, the numbers of slow muscle fibers and muscle pioneers are notably increased (Figure 4G, H). Interestingly, when *dnPKA* mRNA and Dmrt2b-MO are co-injected, the dnPKA can efficiently rescue the development of slow muscle fibers and muscle pioneers in the morphant embryos (Figure 4I, J). Su(fu) acts as a weak inhibitor of the Hh signaling pathway [32], [33]. When Su(fu)-MO is injected into Cont-MO embryos, it leads to number increase of slow muscle fibers, but has little effect on the numbers of muscle pioneers (Figure 4K, L). When Su(fu)-MO and Dmrt2b-MO are co-injected, the Su(fu)-MO can also rescue the development of slow muscle fibers in the morphant embryos (Figure 4M), but the number of muscle pioneer cells can not be increased (Figure 4N). In these experiments, a total of 20 to 38 embryos were contributed to each analysis. Figure 4O and 4P respectively show the quantitative data of slow muscle fibers and muscle pioneer cells per somite. The data indicate that Dmrt2b knockdown does appear to affect slow muscle fibers, and therefore, Dmrt2b is involved in slow muscle development.

### Dmrt2b is involved in neural tube patterning through Hedgehog signaling

**Figure 5:**
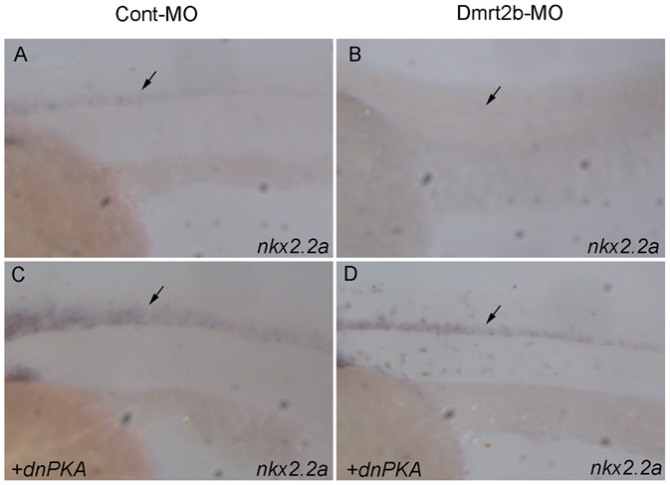
Patterning defects in the neural tube of Dmrt2b morphant embryos. Lateral views of embryos at 24 hpf, anterior to the left, dorsal to the top. (A) Wild phenotype of Cont-MO injected embryo; (B) Severe suppression of *nkx2.2a* in the neural tube of Dmrt2b-MO injected embryo; (C) Ectopic expression of *nkx2.2a* in the neural tube of *dnPKA* injected embryo; (D) The normalized expression of *nkx2.2a* in the neural tube of *dnPKA* and Dmrt2b-MO coinjected embryo. Black arrows indicate the expression position of *nkx2.2a* in the neural tube. All images show the somites over the yolk extension.

Hh signaling has been identified as a key morphogen in patterning of the ventral neural tube [34]. To assess the contribution of Dmrt2b to Hh-mediated signaling in the neural tube, we analyzed the expression of *nkx2.2a*. In non-injected and control-injected embryos, *nkx2.2a* is expressed in lateral floor plate of the neural tube at 24 hpf (Figure 5A). In the Dmrt2b morphant embryos, the *nkx2.2a* expression is severely suppressed (22/25 embryos) (Figure 5B). Moreover, we observed that *dnPKA* mRNA injection could lead to ectopic expression of *nkx2.2a* in the neural tube of Cont-MO embryo (19/25 embryos) (Figure 5C) as described previously [31], [35]. Consistently with the epistatic analysis using slow muscle fiber and muscle pioneer markers (Figure 4I, J), co-injection of Dmrt2b-MO with *dnPKA* normalizes the ectopic expression of *nkx2.2a* (18/26 embryos) (Figure 5D). These data indicate a requirement of Dmrt2b during the development of neural tube for the appropriate cellular response to the Hh signals. To determine whether Dmrt2b is required for transcription of Hedgehog genes, we further examined the expression of three Hedgehog genes *shha*, *ihhb* and *shhb* in Dmrt2b morphant embryos and control embryos. Whole mount *in situ* hybridization analysis showed that *shha*, *ihhb* and *shhb* expression appeared normal in Dmrt2b morphant embryos at 10 hpf (bud stage) and at 24 hpf (Supplementary Figure S2). These data suggest that Dmrt2b should act downstream of Hedgehog genes and upstream of Gli protein, the core components of the Hh pathway [36], because three Hedgehog genes *shha*, *ihhb* and *shhb* are not affected in the Dmrt2b morphants, but *dnPKA* and Su(fu)-MO can counteract the Dmrt2b morphant phenotypes [22], [31].

### Dmrt2b morphants display defects in heart and visceral organs asymmetry

**Figure 6:**
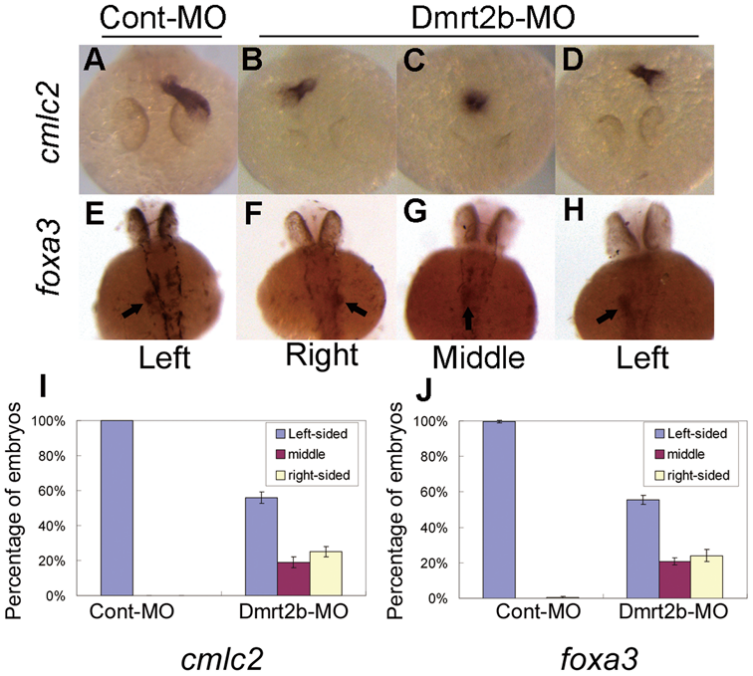
Dmrt2b morphant embryos display defects in heart and visceral organs asymmetry. (A–D) Expression of *cmlc2* in 48 hpf embryos detected by whole mount *in situ* hybridization with antisense RNA probe. (E–H) Expression of *foxa3* in 48 hpf embryos detected by whole mount *in situ* hybridization. Arrows in *foxa3* panel indicate the liver. Graphical representation of the percentage of embryos exhibiting the expression patterns for *cmlc2* (I) and for *foxa3* (J). Results are represented as mean±SD of three separate experiments.

As described previously, Dmrt2a is essential for establishment of LR asymmetry in zebrafish development [14], and *shh* regulates the establishment of LR asymmetry in zebrafish development [37]. We also examined heart asymmetry defects in Dmrt2b morphant embryos by using specific marker of heart, *cardiac myosin light chain 2* (*cmlc2*) [38]. In embryos injected with Cont-MO, 100% of the embryos show the heart tube on the left side, at 48 hpf (Figure 6A, I). By contrast, in the Dmrt2b marphant embryos, 24.9% of the heart tubes shift rightward and 19% remain in the middle (Figure 6B, C, D and I) (n = 86 to 113 in three separate experiments).

In addition to the heart, morphological LR asymmetry of the visceral organs was also observed in zebrafish embryos [37]. To determine if LR asymmetry of the visceral organs was affected in *dmrt2b* knockdown embryos, we checked the expression of *foxa3* which marks the liver. As expected, 100% of embryos injected with Cont-MO exhibit a leftward budding of the liver, at 48 hpf (Figure 6E, J). By contrast, 24.0% of the liver shift rightward, and 20.7% remain in the middle in the Dmrt2b marphant embryos (Figure 6F, G, H and J) (n = 83 to 100 in three separate experiments).

### Dmrt2b activity is important for establishment of left-right asymmetry in lateral plate mesoderm

**Figure 7:**
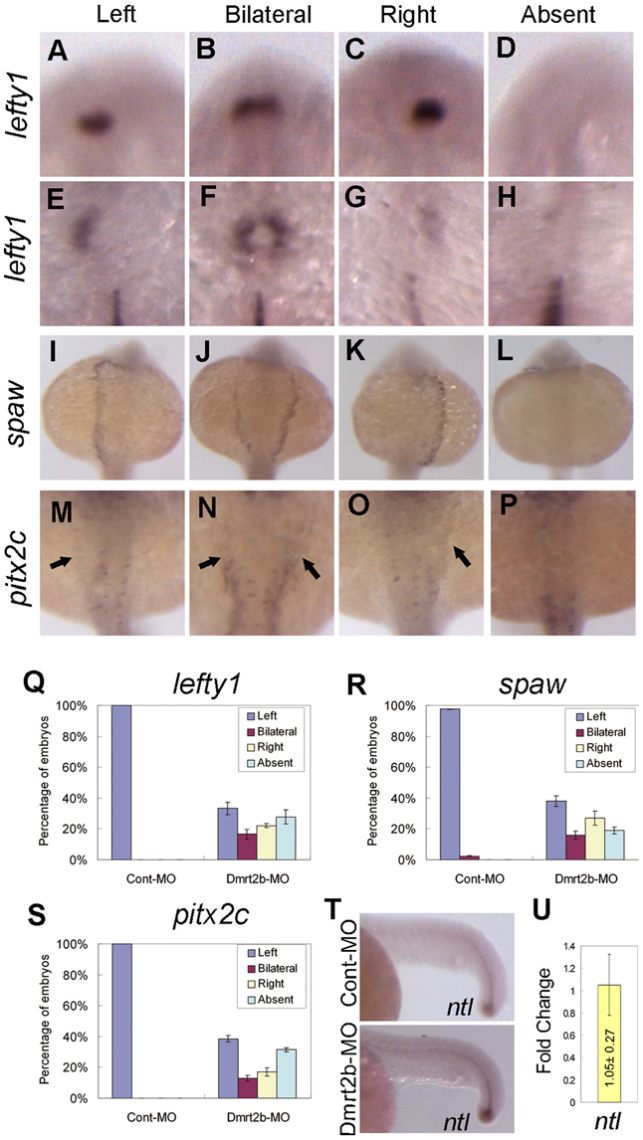
*Dmrt2b* knockdown disrupts L–R identity in lateral plate mesoderm. (A–D) *lefty1* normal expression in left dorsal diencephalon at 22 somites stage were disrupted embryos injected with Dmrt2b-MO. (E–H) *lefty1* normal expression in left LPM at 22 somites stage were disrupted embryos injected with Dmrt2b-MO. (Q) Graphical representation of the percentage of embryos exhibiting the expression patterns for *lefty1* in Dmrt2b knockdown embryos and control embryos. (I–L) *spaw* normal expression in left LPM at 20 somites stage were disrupted embryos injected with Dmrt2b-MO. (R) Graphical representation of the percentage of embryos exhibiting the expression patterns for *spaw* in Dmrt2b knockdown embryos and control embryos. (M–P) *pitx2c* normal expression in left LPM at 22 somites stage were disrupted embryos injected with Dmrt2b-MO. (S) Graphical representation of the percentage of embryos exhibiting the expression patterns for *pitx2c* in Dmrt2b knockdown embryos and control embryos. (T) Lateral view of the embryos at 22 somites stage. Expression of the *ntl* in embryos injected with Dmrt2b-MO is similar to expression of *ntl* in control embryos. Results are represented as mean±SD of three separate experiments. (U) qPCR analysis of the expression changes of *ntl* in 24 hpf embryos injected with Cont-MO or Dmrt2b-MO. The data represents mean±SD of three separate experiments.

Left-sided expression of several genes such as *lefty1, spaw* and *pitx2c* in the lateral plate mesoderm (LPM) is important to heart and visceral organs asymmetry [39], [40], [41]. To determine whether the randomized heart and visceral organs asymmetry is preceded by alteration of underlying molecular cues, we analyzed these LR markers. *lefty1* is a Nodal signaling antagonist, which expresses in the left dorsal diencephalon and left LPM at 22 somites stage (overlapping the prospective heart field) (Figure 7A, E). However only 33.3% of the Dmrt2b-MO injected embryos show the normal *lefty1* patterns, the rest exhibit right-sided, bilateral, or absent expression (Figure 7B–D, F–H and Figure 7Q) (n = 36). *spaw*, which encodes a *nodal*-related protein in zebrafish, is normally expressed in the left LPM at 20 somites stage and regulates left-right asymmetry (Figure 7I). However, the later asymmetric *spaw* expression in the LPM is dramatically altered in Dmrt2b morphant embryos with only 38.1% showing left-sided expression, compared with embryos injected with Cont-MO (Figure 7I–L and Figure 7R) (n = 63). *pitx2c* encoding a bicoid-related transcription factor is also expressed in the left LPM at 22 somite stage (Figure 7M). As expected, in Dmrt2b-MO injected embryos (Figure 7M–P), only 38.6% showing normal left-sided expression compared with embryos injected with Cont-MO (Figure 7S) (n = 70).

Since defects in midline integrity can also lead to abnormal LR asymmetry. We examined the midline structure of these embryos by using *in situ* hybridization of *ntl*. Expression of the *nlt* was not affected in embryos injected with Dmrt2b-MO (Figure 7T and 7U). The above data demonstrate *dmrt2b* importance in the establishment of left-right asymmetry of the body plan.

### Dmrt2a does not contribute to Hedgehog pathway during zebrafish somitogenesis

**Figure 8:**
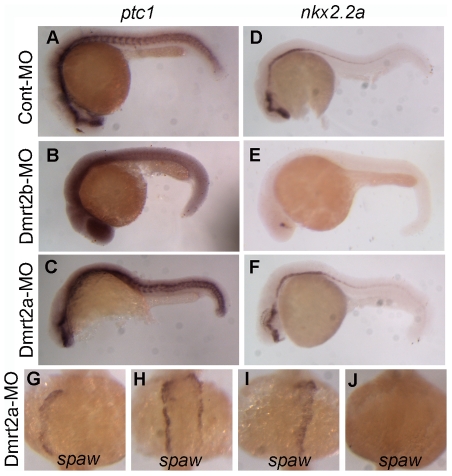
Dmrt2a does not contribute to Hedgehog pathway during zebrafish somitogenesis. (A–C) The expression of *ptc1* in embryos injected with Cont-MO, Dmrt2b-MO and Dmrt2a-MO at 24 hpf. (D–F) The expression of *nkx2.2a* in embryos injected with Cont-MO, Dmrt2b-MO and Dmrt2a-MO at 24 hpf. (G–J) *spaw* normal expression in left LPM at 20 somites stage were disrupted embryos injected with Dmrt2b-MO.

As previously report, Dmrt2a is also involved in somitogenesis in zebrafish [12]. To examine whether Dmrt2a is contribute to hedgehog pathway as Dmrt2b, we performed comparative studies on two marker genes between Dmrt2a and Dmrt2b. As shown in Figure 8, in comparison with normal expression in Cont-MO embryos at 24 hpf (Figure 8A), the down-regulation of *ptc1* transcripts in Dmrt2b morphant embryos (Figure 8B) is not observed in Dmrt2a-MO injected embryos (Figure 8C). Similar results are also observed in checking *nkx2.2a* expression. In contrast with the severely suppression in Dmrt2b morphant embryos (Figure 8E), the expression level of *nkx2.2a* does not reduce in the Dmrt2a morphant embryos (Figure 8F). Moreover, we also detected abnormal *spaw* expression in the Dmrt2a morphant embryos as described previously [14]. Figure 8G–J show the *spaw* expression alteration in left LPM in the 43 Dmrt2a-MO injected embryos with only 39.5% left-sided expression, 20.9% bilateral expression, 23.3% right-sided expression and 16.3% absent expression. The data indicate that Dmrt2a and Dmrt2b play similar rules in the establishment of left-right asymmetry of the body plan, but Dmrt2a does not contribute to Hedgehog pathway during zebrafish somitogenesis.

Dmrt2a has been described to be involved in synchronizing left-right somitogenesis in zebrafish. To clarify whether Dmrt2b has similar role for synchronizing left-right somitogenesis, we further analyzed expression changes of the presomitic mesoderm marker genes *her1* and *deltaC* in the Dmrt2b morphants. As shown in Supplementary Figure S3, the expression of *her1* and *deltaC* are not affected in the Dmrt2b-MO injected embryos. The data confirm that Dmrt2b is not involved in synchronizing left-right somitogenesis in zebrafish.

## Discussion

**Figure 9:**
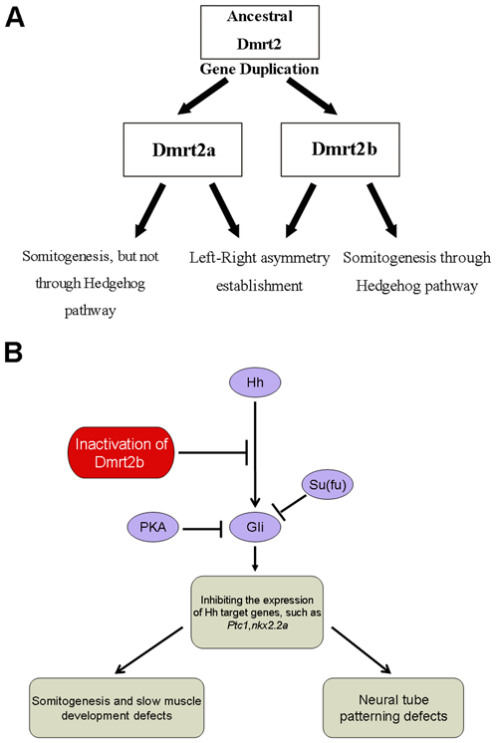
A schematic diagram illustrating the duplication and subsequent functional diversification of zebrafish Dmrt2a and Dmrt2b (A), and a hypothesized acting position that Dmrt2b is involved in Hedgehog pathway (B).

Gene duplication provides raw material for functional divergence and innovation [42], and neofunctionalization and subfunctionalization hypotheses have been proposed. The neofunctionalization hypothesis argues that after duplication one daughter gene retains the ancestral function while the other acquires new functions. In contrast, the subfunctionalization hypothesis asserts that the functions of the ancestral gene are partitioned between the duplicated genes [43]. Recent advances have suggested a new model termed subneofunctionalization, because neither neofunctionalization nor subfunctionalization can alone be explained in a large proportion of duplicate genes, and rapid subfunctionalization is often accompanied by prolonged and substantial neofunctionalization. This new model proposes that a large part of duplicated genes might play both overlapping functions and divergent functions [44]. In this study, we found significant sequence divergence between the fish-specific duplicated gene *dmrt2b* and *dmrt2a*, observed severe defects in somitogenesis, slow muscle development and neural tube patterning in the Dmrt2b loss-of-function zebrafish embryos by using morpholino-mediated knockdown strategy, and revealed the underlying mechanism that is involved in Hedgehog signaling pathway. Moreover, we also found the overlapping functions between *dmrt2b* and *dmrt2a* in LR asymmetry establishments, including heart and visceral organ asymmetry establishment and lateral plate mesoderm asymmetry establishment. Additionally, we further verified that Dmrt2a and Dmrt2b play common overlapping functions in the establishment of left-right asymmetry of the body plan, but Dmrt2a does not contribute to Hedgehog pathway (Figure 9A). Therefore, the current study clarifies exact function of the fish-specific duplicated gene *dmrt2b*, and provides a significant and interesting case for functional divergence of duplicate genes.

Another significant advance in this study was to find similar defect phenotypes in the Dmrt2b morphants to that in the so called “you-type mutants” which are demonstrated to be inactivated in particular genes in the Hedgehog signaling pathway [22], [23], [24], [25], [26], [27], [45]. The defects include the curled tail and the U-shaped somite boundaries. Subsequently, we have confirmed that the expression of Hedgehog target genes *ptc1* and *nkx2.2a* are severely inhibited in the Dmrt2b morphants, and both slow muscle development and neural tube development are also disrupted by Hedgehog pathway impairment. Moreover, we have observed normal expression of three Hedgehog genes *shha*, *ihhb* and *shhb* in Dmrt2b morphants, indicating that Dmrt2b mophant defects are in the response to Hedgehog signals not in Hedgehog transcript expressed at low level. The data suggest that Dmrt2b might act downstream of Hedgehog gene transcription. To assess what level in the pathway Dmrt2b is active, we further used two rescue experiments. Co-injection of *dnPKA* and Su(fu)-MO could rescue some defects of the Dmrt2b morphants in slow muscle development, implicating that Dmrt2b should act downstream of Hedgehog genes and upstream of Gli protein, the core components of the Hh pathway [36], and therefore contribute to somitogenesis, slow muscle development and neural tube patterning by inhibiting the expression of Hh target genes (Figure 9B). In addition, we found that Dmrt2a expression was not affected in the Dmrt2b morphants (Figure 3F and G), and overexpression of *dmrt2a* was previously observed to induce rapid apoptosis in the mesoderm [12]. Moreover, no any Hedgehog signals responding defects were observed in zebrafish Dmrt2a morphants in our experiment (Figure 8). Therefore, the new function of Dmrt2b during somitogenesis and the underlying mechanism that is involved in Hedgehog signaling pathway are different from the function of Dmrt2a during somitogenesis which is linked to apoptosis.

Additionally, an interesting aspect of the Dmrt2b morphants is that it encompasses only a subset of defects observed from other zebrafish Hedgehog pathway mutants. For example, pectoral fins, which are disrupted in mutants *syu* and *smu* [22], [24], appear normal in Dmrt2b knockdown embryos (some Dmrt2b morphant embryos have smaller pectoral fins). We also observed incomplete absence of the slow muscle phenotype. These phenotypic characteristics may be a result of the functionally redundant components in Hh pathway. Indeed, similar varying levels of slow muscle development defects have been described in the different Hh pathway genetic mutants [46], potentially indicating compensatory effects by other genes in the Hedgehog pathway.

In conclusion, we have endeavored to elucidate the roles of Dmrt2b in the developing zebrafish embryo, and compared the Dmrt2b function to the Dmrt2a function. Through morpholino loss-of-function approach, we demonstrated that Dmrt2b contributes to multiple developmental processes, including zebrafish somitogenesis through Hedgehog pathway and establishment of left-right asymmetry. We have uncovered important new roles for Dmrt2b in zebrafish, which were unanticipated by studies of this molecule's duplicated gene Dmrt2a in zebrafish and orthologous gene Dmrt2 in mouse. The data suggest fish-specific duplicated genes Dmrt2b and Dmrt2a play overlapping roles in establishment of Left-Right asymmetry and divergent functions in somitogenesis, which Dmrt2b contributes to Hedgehog pathway.

## Materials and Methods

### Maintenance of zebrafish

A breeding colony of zebrafish (Danio rerio) were maintained at 28.5°C on a 14 h light/10 h dark cycle [47]. All embryos used were collected by natural spawning and staged according to standard procedures [48].

### Antisense morpholino and mRNA microinjection

Two non-overlapping translation-blocking morpholino oligonucleotides (MOs) [16] Dmrt2b-MO1 and Dmrt2b-MO2, standard control MO against the zebrafish β-globin intron, p53 MO, and Su(fu) MO, were obtained from Gene Tools, LLC. The morpholino sequences were as follows: Dmrt2b-MO1, 5′-TTCTCACGAACCCACGACGCTCATC-3′; Dmrt2b-MO2, 5′-CCGCTTTAGTGGACATTTATGAGCT-3′; stand control Mo(Cont-MO), 5′-CCTCCTACCTCAGTTACAATTTATA-3′; p53 MO (p53-MO), 5′- GCGCCATTGCTTTGCAAGAATTG-3′ [20], [21]; Su(fu) MO (Su(fu)-MO), 5′-GCTGCTAGGCCGCATCTCATCCATC-3′ [49]; Dmrt2a MO (Dmrt2a-MO), 5′ - AGATCCGTCATTTTCTGGCCGCGTA – 3′ [14]. Dmrt2b ORF was subcloned into the pCS2+ vector for *in vitro* transcription. *dnPKA* in pCS2+ were kind gifts from Dr. Whitfield (University of Sheffield). Capped sense RNAs were synthesized using the mMESSAGE mMACHINE system (Ambion) from the linearized *dnPKA*-bGFP-pCS2+, and *dmrt2b*-pCS2+ plasmids, re-suspended in 0.1 M KCl. MOs and capped mRNA injections were done into 1-cell-stage embryos according to standard methods. For injection, MOs were diluted at a concentration of about 5 ng each embryo. *Dmrt2b* mRNA were injected at a concentration of about 200pg each embryo. *dnPKA* mRNA were injected at a concentration of about 100 pg each embryo. For co-injection experiment, we injected each embryo twice, first with the MO and then with the mRNA or another MO. After injection, embryos were incubated at 28.5°C in Embryo Medium [47].

### Antibody production

The cDNA fragment coding amino acids 136–364 peptide was subcloned into pET-32a expression vector. A fusion protein was expressed in BL21 (DE3), and detected only in inclusion body. The protein was prepared and immunized mouse as described previously [50].

### Western blotting

Zebrafish embryos were manually dechorionated, deyolked [51] before homogenized. Deyolked samples were dissolved in 2 µl of 2× sodium dodecyl sulfate (SDS) sample buffer per embryo and incubated for 5 min at 95°C. After full-speed centrifugation for 1 min in a microcentrifuge to remove insoluble particles, samples were loaded on a 12% SDS gel. Western blot analysis was performed according to the previous report [50] using the anti-Dmrt2b antibody and anti-tubulin antibody.

### Whole-mount *in situ* hybridization

Antisense probes for *ptc1* [35], *nkx2.2a* [29], *pax6a* [52], *lefty1* [41], *spaw* [39], *cmlc2* [38], *foxa3* [53] and *ntl* were prepared as described previously. Probe for *pitx2c* and *echidna Hedgehog* (*ihhb*) were generated directly from PCR products that included T7 of T3 RNA polymerase binding sequence at the 3′ end. Primer sequences used were: *pitx2c* forward, 5′-ACTGCCGCAAACTTGCATCA-3′ reverse, 5′-CATTAACCCTCACTAAAGGGAAGTTGCTTGGCTTTCAGTCTC-3′; *ihhb* forward, 5′-ATGAGACTCTCCACGGCGGC-3′ reverse, 5′-CATTAACCCTCACTAAAGGGAAATCTCTCAGTTGCCTCTAAG-3′. Probes for sonic Hedgehog (*shha*) and tiggywinkle Hedgehog (*shhb*) were prepared using *shh*-pT7Ts and *twhh*-pT7Ts respectively (kind gifts from Dr. Randall Moon, University of Washington). Embryos were fixed at the stages indicated and processed essentially as previously described [21].

### Immunohistochemistry

Embryos were grown to the indicated stages and processed essentially as described previously. Immunostainings of whole zebrafish embryos were performed following standard protocols. The 4d9 antibody used 1∶100 dulution, which recognizes the *engrailed* protein in the nuclei of muscle pioneer cells, the F59 antibody used 1∶100 dilution, which labels slow myosin heavy clain (MyHC) in zebrafish [30], were obtained from the Developmental Studies Hybridoma Bank. Fluorescent secondary antibodies against mouse used for detection were FITC or Cy3 conjugated. Stained embryos were photographed on fluorescence optics of Confocal Microscope LEICA DMIRE2 (Leica, Germany).

### Quantification of slow muscle defects

As previous report [33], 26 hpf embryos were fixed and stained using F59 antibody. Numbers of slow muscle fibers were counted in 5 somites over the yolk extension per embryo.

## Supporting Information

Figure S1 Dmrt2b translation is blocked by non-overlapping morpholinos. (A) The Dmrt2b-MO1 and Dmrt2b-MO2 target sequences are shown in relation to the 5′UTR region of the Dmrt2b mRNA sequence. (B) Western blot assay showing Dmrt2b translation in the embryos injected with Cont-MO, Dmrt2b-MO1, Dmrt2b-MO2 and Dmrt2b-MO (Dmrt2b-MO1+Dmrt2b-MO2). The signal of Dmrt2b protein were significant reduced in Dmrt2b-MO1, Dmrt2b-MO2 and Dmrt2b-MO injected embryos.
(0.72 MB TIF)

Figure S2 Dmrt2b is not required for shha, ihhb and shhb transcription. Dorsal views of embryos at the bud stage (10 hpf) (A, C, E, G, I and K). Lateral views of embryos at 24 hpf (B, D, F, H, J and L). Expression of shha in embryos injected with Dmrt2b-MO (C and D) is similar to expression of shha in control embryos (A and B). Expression of ihhb in embryos injected with Dmrt2b-MO (G and H) is similar to expression of ihhb in control embryos (E and F). Expression of shhb in embryos injected with Dmrt2b-MO (K and L) is similar to expression of shhb in control embryos (I and J).
(3.61 MB TIF)

Figure S3 Whole-mount in situ hybridization of persomitic mesoderm genes her1 and deltaC in the Dmrt2b morphants. Expression patterns of her1 in the Dmrt2b-MO (A) and Cont-MO (B) embryos. Expression patterns of deltaC in the Dmrt2b-MO (C) and Cont-MO (D) embryos. All the embryos are at 10 somites stage. Panels show dorsal views, anterior to the top.
(4.94 MB TIF)
