# Supplementary figures and images for: Fish-Specific Duplicated dmrt2b Contributes to a Divergent Function through Hedgehog Pathway and Maintains Left-Right Asymmetry Establishment Function
Source: PLoS One. 2009 Sep 30;4(9):e7261. doi: 10.1371/journal.pone.0007261 (PMC2749440; doi:10.1371/journal.pone.0007261)

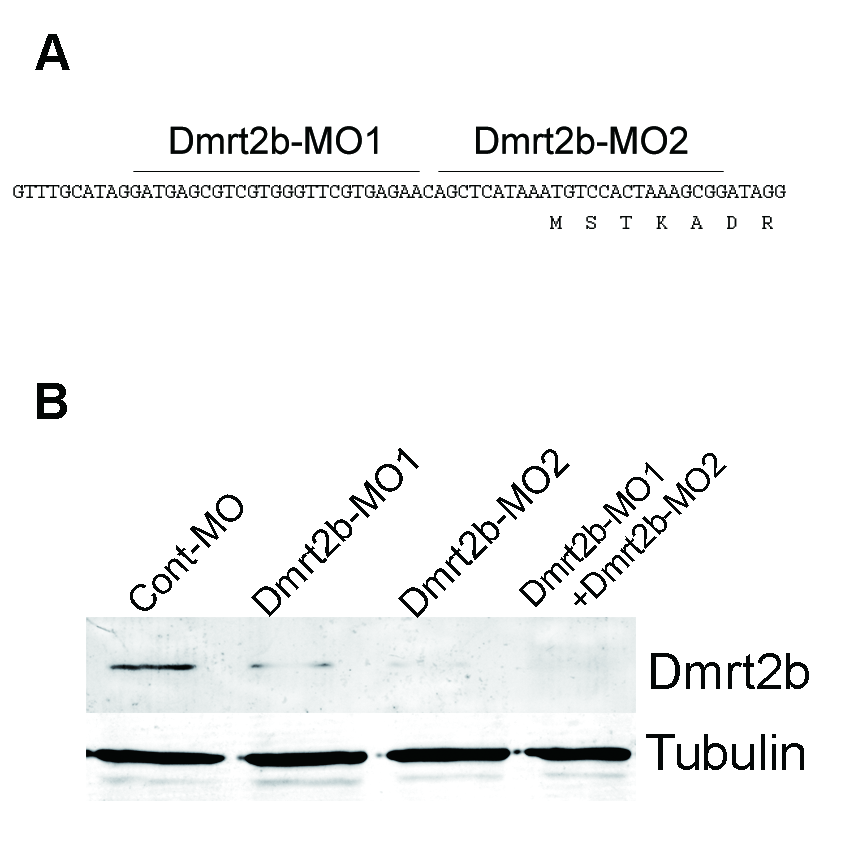

Supplement: Figure S1 — Dmrt2b translation is blocked by non-overlapping morpholinos. (A) The Dmrt2b-MO1 and Dmrt2b-MO2 target sequences are shown in relation to the 5′UTR region of the Dmrt2b mRNA sequence. (B) Western blot assay showing Dmrt2b translation in the embryos injected with Cont-MO, Dmrt2b-MO1, Dmrt2b-MO2 and Dmrt2b-MO (Dmrt2b-MO1+Dmrt2b-MO2). The signal of Dmrt2b protein were significant reduced in Dmrt2b-MO1, Dmrt2b-MO2 and Dmrt2b-MO injected embryos. (0.72 MB TIF) [file pone.0007261.s001.tif]

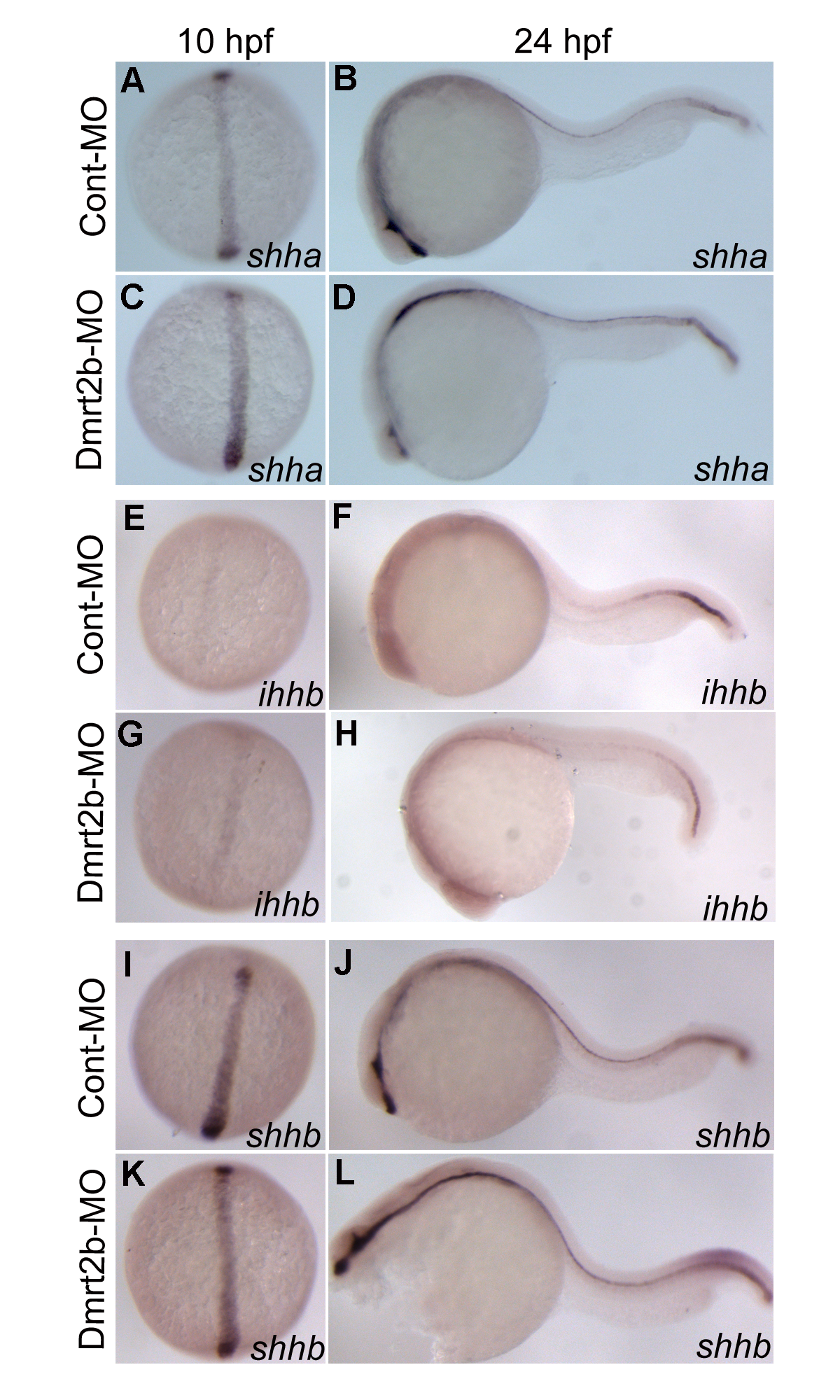

Supplement: Figure S2 — Dmrt2b is not required for shha, ihhb and shhb transcription. Dorsal views of embryos at the bud stage (10 hpf) (A, C, E, G, I and K). Lateral views of embryos at 24 hpf (B, D, F, H, J and L). Expression of shha in embryos injected with Dmrt2b-MO (C and D) is similar to expression of shha in control embryos (A and B). Expression of ihhb in embryos injected with Dmrt2b-MO (G and H) is similar to expression of ihhb in control embryos (E and F). Expression of shhb in embryos injected with Dmrt2b-MO (K and L) is similar to expression of shhb in control embryos (I and J). (3.61 MB TIF) [file pone.0007261.s002.tif]

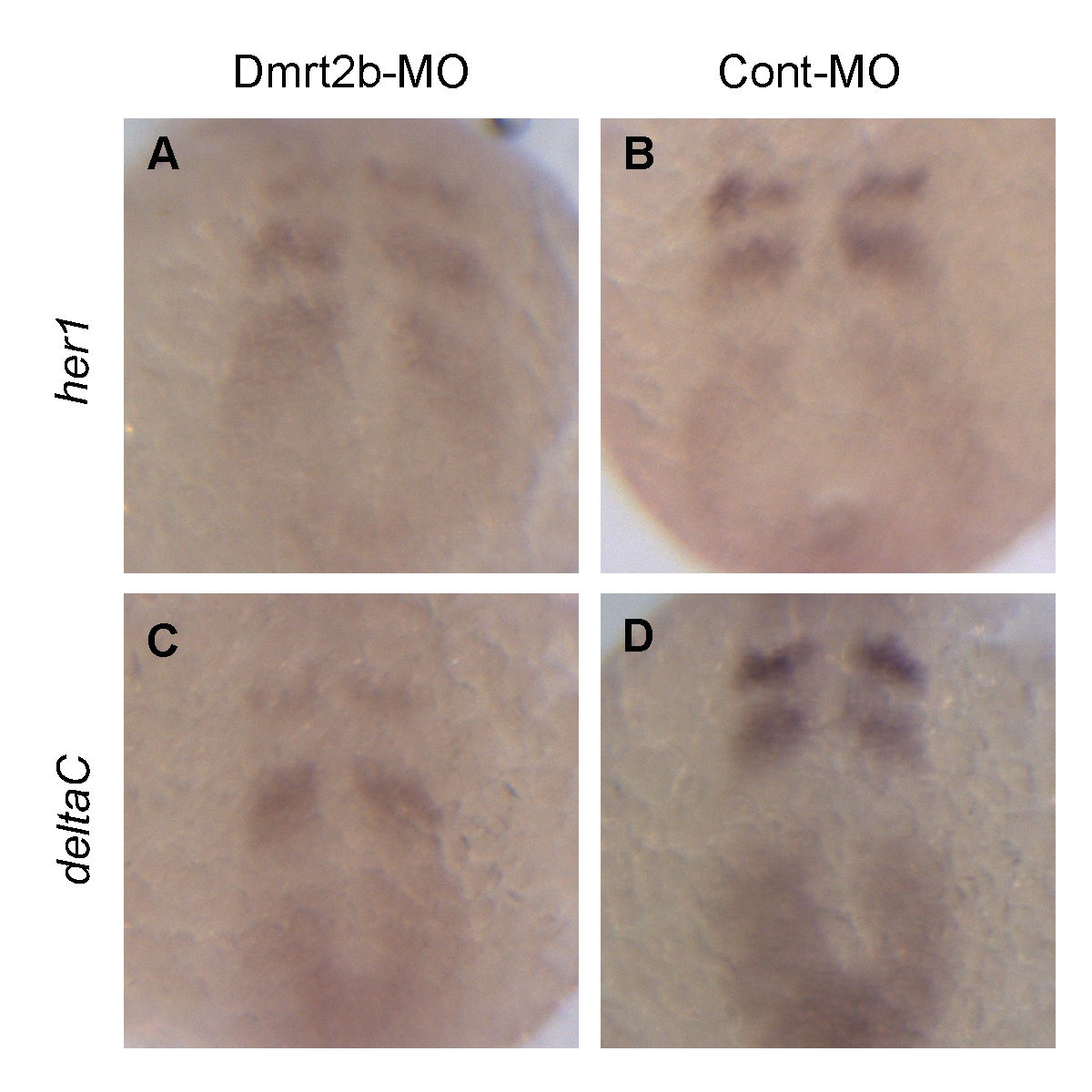

Supplement: Figure S3 — Whole-mount in situ hybridization of persomitic mesoderm genes her1 and deltaC in the Dmrt2b morphants. Expression patterns of her1 in the Dmrt2b-MO (A) and Cont-MO (B) embryos. Expression patterns of deltaC in the Dmrt2b-MO (C) and Cont-MO (D) embryos. All the embryos are at 10 somites stage. Panels show dorsal views, anterior to the top. (4.94 MB TIF) [file pone.0007261.s003.tif]
